# Risk factors for Problematic Internet Use (PIU) among Polish students: the role of loneliness, depression, and health behaviours in a predictive analysis

**DOI:** 10.1186/s12888-026-08098-7

**Published:** 2026-04-27

**Authors:** Marta Kożybska, Iwona Radlińska, Beata Karakiewicz

**Affiliations:** 1 Department of Social Medicine, Subdepartment of Medical Law, Pomeranian Medical University in Szczecin, ul. Żołnierska 48, Szczecin, 71-210 Poland; 2 Department of Social Medicine, Subdepartment of Social Medicine and Public Health, Pomeranian Medical University in Szczecin, ul. Żołnierska 48, Szczecin, 71-210 Poland

**Keywords:** Problematic internet use, Students, Poland, Health behaviors, Depression

## Abstract

**Background:**

In recent years, there has been a revolution in lifestyle and health behaviour. The aim of our study was to examine the relationship between PIU and loneliness, depressive symptoms and health behaviours in a sample of students in Poland.

**Methods:**

A cross-sectional study was conducted among 1,008 Polish students (336 students of medical/health sciences, 336 students of humanities/social sciences, and 336 students of technical faculties). There were 510 women (50.6%) and 498 men (49.4%). The Problematic Internet Use Test (PIU), De Jong Gierveld Loneliness Scale (DJGLS), the Beck Depression Inventory I (BDI I), and the Health-Related Behaviour Inventory (HBI) were used.

**Results:**

Problematic Internet use was observed in 10.2% of individuals. In DJGLS, the respondents scored an average of 25.63 points (out of 55 possible), in BDI I, 28.57% of the participants exceeded the cut-off point of the BDI I test, which may suggest depression, and 53.08% of the respondents showed a low level of health behaviour. Statistically significant correlations were found between PIU and loneliness (rho = 0.226; *p* < 0.001), depressive symptoms (rho = 0.091; *p* = 0.004), overall HBI score (rho = −0.134; *p* < 0.001) and HBI subcategories: positive mental attitude (rho = −0.161; *p* < 0.001), healthy eating habits (rho = −0.089; *p* = 0.005), preventive measures (rho = −0.086; *p* = 0.006) and health practices (rho = −0.086; *p* = 0.007). Linear regression showed that loneliness (*r* = 0.239; *p* < 0.001), depressive symptoms (*r* = 0.066; *p* = 0.037), and positive mental attitude (*r* = 0.079; *p* = 0.084) were predictors of PIU.

**Conclusions:**

The study’s findings indicate that PIU is associated with increases in loneliness, depressive symptoms, and a decrease in health behaviours. In addition, loneliness, depressive symptoms and positive mental attitude were found to be predictors of PIU. These variables should be investigated in prospective studies and included in PIU prevention.

**Clinical trial number:**

Not applicable.

## Background

In recent years, there has been a revolution in lifestyle and health behaviour. The COVID-19 pandemic exacerbated the shift towards online work, study, and daily activities. Studies have shown an increase in the percentage of individuals who are addicted to the internet [1], ], experience loneliness [2], exhibit symptoms of depression [3], or lead unhealthy lifestyles [4]. However, Problematic Internet Use (PIU), loneliness, depression, and negative health behaviours were affecting the overall health functioning of individuals and societies even before the outbreak of the COVID-19 pandemic [5]. According to a recent estimate [6], approximately 14.22% of the global population is addicted to the Internet. Loneliness is experienced by 1.8% to 24.2% of the population over the age of 12, depending on the world region [7]. Additionally, depression affects approximately 5% of adults [8].

Researchers have shown interest in the relationship between PIU, loneliness, and depression. According to the I-PACE model, both loneliness and depression play a significant role in the development of internet addiction [9]. A revised version of the I-PACE model [9] and a recent systematic review on the relationship between loneliness and PIU [10] have also indicated that the relationship is bidirectional. On one hand, individuals experiencing loneliness are more likely to develop problematic internet use (PIU). On the other hand, individuals experiencing PIU are predisposed to experiencing loneliness later [10]. Davis’ cognitive-behavioural model suggests that Internet abuse by individuals experiencing loneliness can be a psychological compensation [11]. Therefore, the Internet can both provide an escape from loneliness and contribute to it due to reduced real-world social contacts. Experiencing loneliness increases the risk of depression [12]. Symptoms of depression often co-occur with excessive Internet use [10]. It has been speculated that the Internet may be a coping strategy to deal with negative feelings or stressful situations for people with depressive symptoms [13].

In recent years, there has been a reported increase in internet addiction [1], accompanied by a decline in health behaviours. Researchers report worsening eating habits [14] and reduced levels of physical activity [4]. Numerous studies have found associations between PIU and unhealthy lifestyles. Regarding dietary behaviour, research suggests that PIU is linked to overweight and obesity [15]. Additionally, a correlation has been found between the amount of time spent studying online and the consumption of sugary drinks and skipping breakfast [16]. PIU is also associated with lower levels of physical activity [17]. Furthermore, individuals with PIU are more likely to abuse psychoactive substances and engage in gambling [18]. According to research, these individuals are also more likely to experience sleep disturbances [19].

Both mental well-being and PIU are linked to an individual’s health behaviours. For instance, individuals with depression are more likely to have unhealthy lifestyles, such as smoking, low physical activity, obesity, and dyslipidemia [12]. Moreover, those who experience a decline in mental health also tend to make poor dietary choices and reduce their physical activity [14]. Physical activity and a healthy lifestyle, in general, promote psychological well-being. Studies suggest that physical activity can prevent and alleviate internet addiction [20].

Given the rising number of individuals with internet addiction, it is crucial to investigate the associations between PIU and psychosocial functioning and lifestyles. The harms associated with online activity are increasingly recognised as a societal problem requiring multisectoral public health interventions, involving governments, educational institutions, technology companies, and healthcare systems [21]. Given the widespread use of digital technologies and the adverse effects associated with their misuse [21], researchers are emphasising the importance of attitudes and behaviours that promote mindful use. This suggests that digital well-being could be considered an important aspect of quality of life [22]. Researchers are investigating the traits and characteristics of individuals who use digital tools in ways that promote their well-being [23]. This includes consciously restricting technology use, for example through digital detox [24] or experiencing the ‘joy of missing out’ [25]. A meta-analysis by Lo et al. indicates that addressing a specific issue such as PIU can lead to improvements in quality of life across various domains, including overall health [26]. At the same time, emerging evidence on technostress [27] and psychological distress [28] highlights the need to consider broader psychosocial mechanisms underlying PIU. These findings suggest that lifestyle and mental health factors may be involved in relationships with PIU, underscoring the importance of further research on these associations. This is particularly essential for developing effective prevention and treatment programmes for individuals with Internet addiction [29]. A comprehensive understanding of the mechanisms and factors associated with PIU can aid in the planning of intervention programmes that address multiple constructs simultaneously, such as PIU and loneliness [10].

To the best of our knowledge, no studies have been conducted on the association between PIU, loneliness, depressive symptoms, and health behaviours in a large sample of students with proportional representation by gender and field of study. Previous studies among young adults have included small samples [14, 16, 30–32], students of only one discipline, with a predominance of medical students [5, 33–37], or have not linked constructs of mental functioning to health behaviours [38]. Of note is the study by Wang et al. on the role of loneliness and learning burnout in the regulation of physical activity in internet addiction [20]. However, they were conducted on a Chinese population and only capture one aspect of health behaviour, namely physical activity. The results of Bener and Bhugra [39] are also very interesting. They showed that there is an association between PIU and negative lifestyle, and risk factors for depression. This study was carried out in a Middle Eastern population and needs to be tested in other parts of the world. There is considerable social, economic and cultural variation in PIU [40, 41]. For this reason, research from one country may not be applicable to other countries [42], so results obtained in Asia or the Middle East may not be confirmed in Europe.

To fill this gap, the aim of our study was to examine the relationship between PIU and loneliness, depressive symptoms and health behaviours in a sample of Polish students with proportional representation by gender and field of study. It was decided to examine health behaviours in a holistic way, taking into account eating habits, preventive behaviours (following medical recommendations, avoiding illness), positive mental attitude (avoiding highly stressful and depressing situations) and health practices (sleep, recreation, physical activity), according to Gochman’s approach [43, 44].

Based on this research objective and the current state of knowledge, the following research hypotheses were adopted:

1. Loneliness is a predictor of PIU.
2. Depressive symptoms are a predictor of PIU.
3. Low levels of health behaviours related to dietary habits, preventive behaviours, positive mental attitudes, and health practices are predictors of PIU.

## Methods

### Study procedure

This article presents the third part of the results of the project ‘Health risks associated with problematic Internet use’ [45, 46]. A cross-sectional study was conducted to verify the hypotheses. The study was approved by the Bioethics Committee of the Medical University of Pomerania in Szczecin (Decision No: KB-0012/188/05/17) and carried out in November and December 2018 in Poland. We decided to include students of public universities from different cities (Lublin, Katowice, Gliwice), with different profiles of education: medical, technical, and economic. The inclusion criterion was the status of a full-time student, first-cycle studies, or 1st, 2nd, or 3rd year of single-cycle studies (applicable to medical and dental degrees). We included lower-year students because they are at higher risk of PIU [47]. Because we wanted to examine PIU among students of various fields, the inclusion criterion was also studying medical and health sciences or humanities and social sciences or technical faculties. We aim to include a comparable number of women and men from each university and each group of study fields, so gender was also a criterion for inclusion. The sample size was calculated to be 663 (using the EPI InfoTM 7.2.4.0; the Centers for Disease Control and Prevention, Atlanta, Georgia, USA). The incidence of PIU was estimated to be 10% based on the literature available at the time of study design [48]; the number of full-time undergraduate students in Poland was 895.725 [49], the confidence level was 99.0%, confidence limits 5% [46]. To ensure the best quality of data, we decided to collect 1008 completed questionnaires:

1. 336 from medical and health science students, with equal participation of women and men;
2. 336 from humanities and social sciences students, with equal participation of women and men;
3. 336 from students of technical faculties, with equal participation of women and men [45, 46].

The study was conducted by the professional research agency Grupa BST Sp. z o.o. at three universities in Poland with the approval of the university authorities. The study involved students from the Medical University of Lublin, the Silesian University of Technology in Gliwice, and the University of Economics in Katowice. The study was conducted by trained interviewers. The paper-and-pencil method was used. The interviewer met with students after classes and students could voluntarily and anonymously complete the survey. Those who did not want to take part in the study could freely leave the room. This study used an implied consent procedure, following consultation with the Bioethics Committee of the Pomeranian Medical University in Szczecin. The Committee confirmed that, due to the non-interventional and anonymous survey design, the study did not meet the criteria of a medical experiment or clinical trial and did not require written consent (Decision No: KB-0012/188/05/17). Participants were informed about the purpose of the study and the possibility of withdrawing from it at any stage. Participants were informed that completing the survey constituted implied consent to participate in the study. According to this procedure, implied consent was obtained from all subjects taking part in the study. This approach allowed us to uphold the principle of minimizing the collection of personal data while ensuring ethical participation in the study. A total of 1,008 correctly completed questionnaires were collected. There were 510 women (50.6%) and 498 men (49.4%). The age range was 18–40 years (SD = 2.65), with a mean age of 21.3 years. The details of the study design, sample size, study procedure, and inclusion and exclusion criteria have been described in previous publications [45, 46].

### Participants

A cross-sectional study was conducted at three universities with different educational profiles (medical, technical, and economic) in Poland. The sample size was calculated to be 663 [46]. The analysis included data from 1,008 students in their first three years of study. The mean age of the participants was 21.3 years, and 50.6% were women [45, 46]. Detailed socio-demographic characteristics are presented in Table 1.

**Table 1:** Socio-demographic characteristics of the study participants

	n/M	%/SD
***Gender***		
women	510	50.6
men	498	49.4
***Age (range 18–40)***	**21.31**	**2.65**
*Medical and health sciences students* women men	*336* 174 162	*33.3* 17.3 16.1
***Science/technical students*** women men	*336* 168 168	*33.3* 16.7 16.7
***Humanities/social sciences students*** women men	*336* 168 168	*33.3* 16.7 16.7

### Measures

#### Problematic internet use test TPUI22 (PIU)

The Polish adaptation [50] of the Internet Addiction Test (IAT) developed by Dr Kimberly Young [51] was used to measure problematic Internet use. The characteristics, structure, and interpretation of the Polish questionnaire were described in detail in the previous publication of the first part of our results [46]. This questionnaire contains 22 questions, the answers to which are constructed on a Likert scale (from 0=‘never’ to 5=‘always’). The higher the score obtained, the higher the severity of problematic Internet use (maximum score is 110 points). The score obtained can be assigned to one of the following categories: very low risk of internet addiction, low risk of internet addiction, moderate risk of internet addiction, high risk of internet addiction, and very high risk of internet addiction. The cut-off for the high and very high risk of internet addiction categories is 50 points for individuals aged ≤ 24 years and 42 points for those aged > 24 years. The score ranges for each category are presented in a previous paper [46]. The tool is reported to have good reliability (Cronbach’s alpha = 0.935) [52].

#### Beck depression inventory I (BDI I)

The study employed the Polish adaptation of the Beck Depression Inventory I (BDI I) to measure symptoms of depression. A higher score indicates more severe depressive symptoms. The cutoff point for diagnosing a depressive episode is 12 points out of a possible 63. The Cronbach’s alpha coefficient is 0.87 [53].

#### De Jong Gierveld loneliness scale (DJGLS)

To measure loneliness, the De Jong Gierveld and Kamphuis scale was used [54], in the Polish adaptation by Grygiel et al. [55]. The scale comprises 11 statements with five response categories: ‘yes!’, ‘yes’, ‘more or less’, ‘no’, and ‘no!’. It contains six negative items (2, 3, 5, 6, 9, 10) that describe dissatisfaction with social contacts and five positive items (1, 4, 7, 8, 11) that relate to satisfaction with interpersonal relationships. The scale ranges from 1 (‘strongly agree’) to 5 (‘strongly disagree’). The negative items should be recoded and summed with the positive items. Higher scores indicate greater feelings of loneliness. There is no specific cutoff point. The authors of the adaptation suggest presenting the total scale scores without dividing them into subcategories. The Cronbach’s alpha is 0.89 [55].

#### Health-related behaviour inventory (HBI) by Zygfryd Juczyński

The Polish standardised Health-Related Behaviour Inventory (HBI) by Zygfryd Juczyński was used to measure health behaviours. The scale contains 24 questions on health behaviours, which the respondent answers on a scale from 1 (‘almost never’) to 5 (‘almost always’). The result is calculated by summing the points. A higher score means a higher frequency of health behaviours. The raw score can be interpreted as a low (24–71 for men and 24–77 for women), medium (72–86 for men and 78–91 for women) or high (87–120 for men and 92–120 for women) intensity of health behaviour. The Cronbach’s alpha for the whole scale is 0.85. In addition to providing an overall score, the tool distinguishes four categories of health behaviour:

1. Healthy eating habits (e.g. questions about eating fruit and vegetables, wholesome bread, limiting animal fats, sugar, salt, preservatives).
2. Preventive behaviours (avoiding diseases, following medical advice, regular medical check-ups, seeking information on health and illness).
3. Positive mental attitude (avoiding depressing situations, avoiding stress and tension, having friends and a regulated family life, avoiding negative feelings, thinking positively).
4. Health practices (leading a healthy lifestyle, questions about rest, sleep, weight control, smoking, avoiding overly excessive exercise).

Each subscale comprises six items, and its score is calculated as the sum of the points assigned to these items.

The Cronbach’s alphas for the four subscales individually remain in the range from 0.60 to 0.65 (62).

#### Self-designed socio-demographic and internet use characteristics survey

The respondents were asked questions about their sociodemographic variables (gender, age, field of study, self-reported financial status, extracurricular activity, and amount of free time) and characteristics of Internet use (main device, primary purpose, and screen time for study and other activities). The construction of the tool was presented in a previous article [45].

### Statistical analysis

Statistical analysis was performed using the IBM SPSS Statistics package and IBM SPSS Amos v. 25. The licences for these programs are held by the professional statistician who performed the analyses. A significance level of *p* < 0.05 was used. The chi-square and Kolmogorov-Smirnov tests were used to evaluate the distributions of qualitative and quantitative variables. Additionally, Spearman and Pearson correlation analyses and multivariate regression analyses were performed using a linear model. Due to deviations from the assumptions of normal distributions, a Bootstrapping procedure was used with a maximum draw of 1,000 subgroups.

## Results

The socio-demographic and Internet use characteristics of the study group have been described in detail in a previous publication [45].

Table 2 presents the distributions of scores for PIU, sense of loneliness (DJGLS), depressive symptoms (BDI I), and health behaviour (HBI). All distributions were significantly different from normal. The PIU measure scores exhibited high skewness, with the majority of the participants scoring below the group mean. On average, the participants scored 25.84. As detailed in our previous publication [46], 70.5% of the participants had an average risk of internet addiction. Problematic Internet use was observed in 10.2% of individuals.

**Table 2:** Basic distributions of PIU, BDI I, DJGLS, HBI scores

Variables tested	Min	Max	M	SD	Mdn	Skewness	Kurtosis	Normality of the distribution
PIU	0.00	110.00	25.84	20.35	21.00	1.692	3.270	<0.001
BDI I	0.00	56.00	8.16	8.47	6.00	1.543	3.192	<0.001
DJGLS	11.00	51.00	25.63	8.24	25.00	0.157	−0.619	<0.001
HBI	24.00	120.00	72.56	15.62	73.00	−0.307	0.748	<0.001
HBI healthy eating habits	6.00	30.00	17.72	4.59	18.00	0.057	−0.001	<0.001
HBI preventive behaviours	6.00	30.00	18.02	4.57	18.00	−0.054	−0.015	<0.001
HBI positive mental attitude	6.00	30.00	18.87	4.99	19.00	−0.264	−0.400	<0.001
HBI health practices	6.00	30.00	17.94	4.56	18.00	−0.073	−0.017	<0.001

Min, Minimum; Max, Maximum; M, Mean; SD, Standard deviation; Mdn, median; PIU, Problematic Internet Use Test; DJGLS, De Jong Gierveld Loneliness Scale; BDI I, Beck Depression Inventory I; HBI, Health-Related Behaviour Inventory

In the measurement of loneliness (DJGLS), individuals with moderate levels were found to occur with similar frequency to those with low and very low levels of loneliness (low kurtosis). The respondents scored an average of 25.63 points (out of 55 possible). The BDI I measure was dominated by individuals scoring lower than the group average in depressive severity (high kurtosis). The participants scored an average of 8.16 points. 28.57% of the participants exceeded the cut-off point of the BDI I test, which may suggest depression (Table 2).

In the health behaviour measures, a larger proportion of individuals obtained an average score rather than an extreme score, resulting in high kurtosis. In the overall HBI score, the respondents received an average of 72.56 points (out of 120 possible points). Analysing the categorical score, 53.08% (535) showed a low level of health behaviour; 34.62% (349) had a medium intensity of health behaviour, while 12.30% (124) showed a high rate of health behaviour. In the HBI subcategory of healthy eating habits, the respondents scored an average of 17.72 out of a possible 30 points. In the preventive behaviour subcategory, they scored 18.02, in the positive psychological attitude subcategory, 18.87, and in the health practices subcategory, they scored 17.94 out of a possible 30 points (Table 2).

All variables were significantly correlated with each other, although the correlations were weak. The strongest correlation was found between PIU and feelings of loneliness (rho = 0.226; *p* < 0.001). A weaker correlation linked PIU with the severity of depressive symptoms (rho = 0.091; *p* = 0.004). Both correlations were positive, which indicates that with the increase in PIU, increases in both the sense of loneliness and the severity of depressive symptoms were observed. The associations between PIU and health behaviours were also weak but negative. While the severity of PIU increased, general health behaviours (rho = −0.134; *p* < 0.001), positive mental attitude (rho = −0.161; *p* < 0.001), healthy eating habits (rho = −0.089; *p* = 0.005), preventive measures (rho = −0.086; *p* = 0.006) and health practices (rho = −0.086; *p* = 0.007) decreased (Table 3).

**Table 3:** Spearman’s correlation coefficients between PIU and DJGLS, BDI I, HBI scores

Factors	*r*_*s*_	p
BDI I	0.091	0.004
DJGLS	0.226	<0.001
HBI	−0.134	<0.001
HBI healthy eating habits	−0.089	0.005
HBI preventive behaviours	−0.086	0.006
HBI positive mental attitude	−0.161	<0.001
HBI health practices	−0.086	0.007

PIU, Problematic Internet Use Test; DJGLS, De Jong Gierveld Loneliness Scale; BDI I, Beck Depression Inventory I; HBI, Health-Related Behaviour Inventory; r_s_, Spearman’s rank correlation coefficient; p, significance

**Fig. 1:**
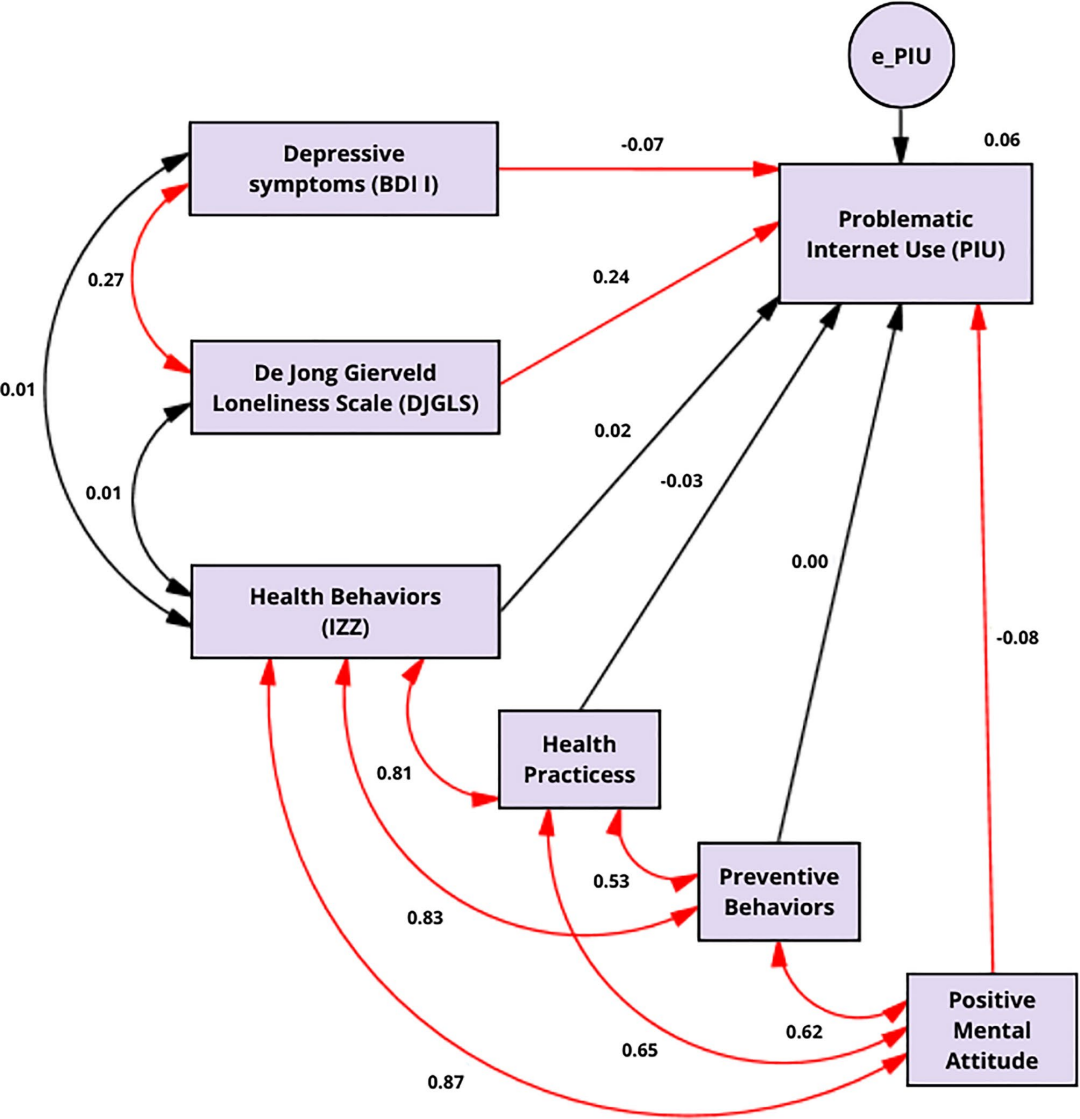
Dependency prediction model - PIU, loneliness, depressive symptoms, and health behaviours

A linear regression method was used to test whether PIU severity could be predicted by feelings of loneliness, depressive symptoms, and health behaviours, and whether the explanatory variables were related to each other. Figure 1 shows the tested relationship model, which had a good fit (CLI = 0.925).

The HBI subcategory of healthy eating habits was excluded from the model due to its lack of a linear relationship with PIU and its significant negative impact on the model fit. The most significant predictors of PIU were feelings of loneliness – for every 1-point increase in the DJGLS score, there was a 0.239 standard deviation increase in the PIU measure (*p* < 0.001). A significant but slightly weaker association was found regarding the severity of depressive symptoms. The relationship was negative, indicating that for every 1-point increase in BDI I score, there was a decrease in PIU score by 0.066 standard deviations (*p* = 0.037). Among the health behaviour indicators, only a positive mental attitude was found to be associated with PIU, at a level of the statistical trend that was not fully significant. A 1-point increase in this scale score was accompanied by a 0.079 standard deviation decrease in PIU (*p* = 0.084). Health behaviours did not show any correlation with either loneliness or depressive symptoms. However, an increase in feelings of loneliness was found to be associated with an increase in the severity of depressive symptoms (*r* = 0.273; *p* < 0.001).

## Discussion

### PIU and loneliness and depression

Our study showed a positive correlation of PIU with feelings of loneliness and with symptoms of depression. Both feelings of loneliness (positive correlation) and symptoms of depression (negative correlation) appeared to be predictors of PIU. The link between loneliness and PIU has been confirmed by other researchers, mainly in youth populations (adolescents and young adults), the most active age group online [10].

Other studies have shown that PIU can lead to alienation and feelings of loneliness by limiting real-world relationships, which are replaced by online contacts [56, 57]. It should be noted that according to the findings of a 2024 systematic review, the links between PIU and loneliness are often bidirectional, with loneliness being both a cause and a consequence of PIU [10]. Both factors can also create a vicious circle and reinforce each other (e.g. loneliness can lead to PIU, which will further exacerbate loneliness, or vice versa, PIU leads to loneliness, which leads to increased PIU, etc.) [10]. This bidirectional relationship has been demonstrated both in theoretical models such as Barnd et al.’s I-PACE Model (Person-Affect-Cognition-Execution) [9] and in empirical research [10].

Research has shown that the use of the Internet in leisure time for entertainment or relaxation, rather than as a tool for work or study, has the greatest impact on the development of PIU [5, 45]. Since everyone has limited free time, engaging in online recreation often requires sacrificing other activities, such as spending time with family and friends. This leads to a reduction in real-life interpersonal contact, which may result in a greater desire for online contact. However, online contact is always a substitute for face-to-face contact and cannot replace it in terms of feelings of loneliness [58]. Online activity can limit the establishment of new relationships and building lasting ties in one’s community (e.g. a young person with a phone may ignore their peers on the way home from university and not spend free time with them). The described mechanism of the self-reinforcing spiral of loneliness – PIU, PIU – loneliness is unfortunately becoming increasingly common among young people and young adults [10].

Our study showed a weak positive correlation between depression symptoms and PIU. Interestingly, the association between these variables was negative – an increase in depression symptoms was associated with a decrease in PIU symptoms. This unexpected finding may be explained by the fact that depression is a heterogeneous illness. It may be that some people escape into the virtual world, while others withdraw from social life, both real and virtual, and limit the time they spend online. It should also be noted that for a third of the group studied, social communication was the main purpose of using the Internet, as indicated in our previous article [45]. Other authors have obtained results indicating a positive association – depression significantly and positively predicted PIU [59]. Most studies suggest that depression exacerbates PIU symptoms [57, 60–66]. Some authors have shown a bidirectional relationship between PIU and depressive symptoms [66].

Our study also found that feelings of loneliness were correlated with depressive symptoms, and that as the feeling of loneliness increased, the symptoms of depression also increased. This correlation is supported by studies by other authors [59]. Recent studies also suggest that this may be a bidirectional interaction [67, 68]. It should be added that population-based studies in this area most often focus on young people, including adolescents, and the elderly, who tend to cope worst with loneliness.

### PIU and positive mental attitude

Our study was one of the first to include a wide range of health behaviours as predictors of PIU, in addition to depression and loneliness. We found that both the overall HBI score and its categories were negatively correlated with PIU. The higher the PIU score, the lower the level of health behaviour. Only a positive mental attitude, understood as optimism (positive thinking), avoidance of difficult situations, tension, strong emotions, stress, and having social ties (friends, family), was a predictor of PIU.

A strength of our study is the use of a standardised tool to measure health behaviour holistically. Unfortunately, we can only make a limited comparison with the results of other researchers due to the wide variety of mostly non-standardised health behaviour measurement tools used by other authors. Each country used its tools, which grouped and named the health behaviours slightly differently. The health behaviours used in the Polish HBI scale correspond most closely to scales related to lifestyle and well-being and, in the context of positive mental attitude, to optimism and self-esteem (optimism, self-esteem). The authors of the 2023 systematic review identified both depression and mental attitude-related factors among the risk factors for PIU [59]. Hinić et al. showed an association between positive life orientation (i.e., life satisfaction, positive self-esteem, and optimism as its indicators) and PIU. Pessimism was the most important individual variable explaining the increase in PIU [69]. In an Italian study, Casale et al. demonstrated that low psychological well-being is associated with PIU [70]. The results of a systematic review suggest the PIU has a significant and negative effect on well-being [71]. Thus, the results of previous studies indicate a similar relationship of PIU with the mental component, as in our study. These results suggest that attention to mental well-being and mental health promotion may prevent PIU, but this needs to be confirmed in prospective studies.

### PIU and other health behaviours

In our results, both the total HBI score and all its categories were negatively correlated with PIU. Health behaviours, apart from the positive mental attitude category, were not a predictor of PIU.

There are few studies in the literature on the general health behaviour index compared to PIU. The study by Duran and Alemdar is noteworthy. They used the Family Nutrition and Physical Activity Tool, a standardised nutrition and physical activity questionnaire that measures the regularity of eating habits and physical activity, to determine whether children’s family environment, practices, and behaviours pose a risk for developing obesity. The authors surveyed children aged 7–10 and obtained a statistically significant, but very weak, negative correlation between The Family Nutrition and Physical Activity Tool score and internet addiction. However, the authors point out that the majority of the children in the study were of normal weight, and further analysis showed that internet addiction was a significant risk factor for obesity. This meant that the increase in internet addiction was accompanied by the children’s unhealthy eating habits and low levels of physical activity [72]. While there is a lack of research in the literature on the association of overall health behaviour scores with PIU, other researchers have confirmed the association of PIU with individual health behaviours. To date, the most commonly studied health behaviour variables are dietary habits and physical activity.

### Eating habits

One of the categories of the HBI questionnaire that we use is correct eating habits. This category includes questions about eating fruit and vegetables, eating wholemeal bread, and limiting sugar and animal fats, salt, and preservatives. Correct eating habits are negatively correlated with PIU. This is in line with reports from other researchers. A study of the Turkish population aged 15–24 years used the Nutrition Exercise Behaviour Scale, which assesses dietary habits and physical activity levels simultaneously. The authors found a positive correlation between internet addiction and unhealthy eating habits. In addition, the tool used allowed the authors to show a correlation between internet addiction and the psychological eating behaviour subcategory, which measures addictive eating behaviour [73]. Similar relationships between psychological eating behaviour and internet addiction have also been shown by other authors [74]. In a group of children aged 7–10, a link was found between internet addiction and eating while using the Internet [75]. This behaviour can negatively affect the quantity and quality of food consumed. Among Thai students, 34.5% were shown to consume snacks while studying online. Ready-to-eat savouries, bakery good and confectionery items were the most popular choices [16].

The link between unhealthy eating habits and internet addiction has also been shown by other researchers, for example from Korea and Europe [76, 77]. Digital hygiene rules should include guidelines for eating balanced meals on a regular basis, without using the Internet while eating.

### Preventive behaviour

Preventive behaviour as a category of HBI includes avoiding illness, following medical recommendations, having regular medical check-ups, and seeking information about health and illness. In our study, this category was negatively correlated with PIU. Seeking information about health and disease is part of health awareness. This awareness should lead to health-promoting behaviours, including preventive care, keeping medical appointments, and following doctors’ recommendations. Today, the Internet is the most common way to search for health information.

A study by Hills & Shah [78] found that searching for information on health-related websites was associated with more timely check-ups and more positive beliefs about preventive medical care among African Americans. However, if the repeated or excessive search for health-related information is associated with distress, it can exacerbate health anxiety, which is called ‘cyberchondria’. In a study by Fegrus & Dolan, respondents who experienced increased health anxiety after searching for medical information on the Internet reported significantly higher PIU [79]. Studies by many authors suggest that cyberchondria is strongly associated with PIU [80, 81]. Regarding other preventive health behaviours, a study by Peltzer et al. found an association between heavy Internet use and a lack of dental check-ups [18].

### Health practices

In our study, health practices included behaviours such as getting enough rest, avoiding overwork, controlling body weight, and avoiding smoking. The total score on this scale was negatively correlated with PIU. A study conducted in Thailand on a sample of 860 students showed that a sedentary lifestyle, abnormal body weight (too high and too low), use of illicit drugs and gambling were among the health practices associated with PIU [18]. The most recent meta-analyses confirm the associations of PIU with overweight and obesity [15], as well as with sleep disturbances [19].

Another very important health practice is physical activity. Previous studies have often examined this variable, and the results obtained indicate that there is a positive correlation between low levels of physical activity and internet addiction [17]. Cigarette smoking [82] and psychoactive substances use [18] are other negative health practices correlated with internet addiction.

The results of our research indicate that good dietary habits, preventive behaviours, and health practices are not predictors of PIU. A small number of studies suggest that selected health behaviours are not associated with PIU at all. A surprising study in a Taiwanese population found that problematic social media use and problematic gaming were associated with increased physical activity (as opposed to smartphone use) [83]. Similar unexpected results were found in a large Asian sample (3,135 mainland Chinese, 600 Taiwanese, and 622 Malaysians) – greater nomophobia (fear of losing a mobile phone) was associated with higher levels of physical activity [83].

In our study, health behaviours, as measured by the HBI inventory, were not correlated with either loneliness or depression. In studies by other authors, lifestyle and well-being, as measured by the Lifestyle and Well-Being Index tool (LWB-I, which includes: family history of disease, pre-existing disease, smoking status, insomnia, physical activity, eating vegetables and fruits, adding sugar) were correlated with depression [84, 85]. Other work shows that loneliness is also associated with lifestyle [86], for example in a Swedish study (assessed by the criteria: smoking status, chronic alcohol consumption, binge drinking, physical activity, diet awareness) [87]. However, both in the study by Cuesta-Lozano et al. and in our study, there was no association between lifestyle (assessed by dietary behaviour, physical activity, smoking, and drinking status) and loneliness [88]. These differences in results may be due to the use of different tools and the broader understanding of health behaviours in our research (as described in the methodology).

According to a recent literature review, online activity can have a good impact on well-being and lifestyle, as long as it facilitates the management of health behaviours and does not have PIU characteristics [89]. Self-management of health behaviour using the Internet can be done through membership in groups interested in healthy lifestyles, consultations, and expert advice, as well as using calendars to plan nutrition, physical or cultural activities. Of particular importance is the use of e-health applications that allow online registration, reminders of medical appointments, or the need to purchase e-prescriptions. These apps also have many other features, such as heart rate monitors, step counters, nutrition counters, QR code scanners for groceries, etc. A theoretical model has even been proposed: the Compensatory Carry-Over Action Model (CCAM), which describes the mechanism of using the Internet to maintain lifestyle and well-being behaviours [89]. Such applications can alert the user if there is a need to pause the use of an application, warning against excessive internet use.

Future research should employ longitudinal designs to clarify the causal relationships between PIU, loneliness, depressive symptoms and health behaviours, paying particular attention to positive mental attitude. Studies should also control for key psychological factors, such as self-efficacy, self-esteem, emotion regulation, coping styles, impulsivity and perceived stress, as these may act as important mediators or moderators of these associations

## Limitations

Although our research has increased knowledge of predictors for PIU, it is not without limitations. Participants were purposively selected according to their field of study, allowing for an equal distribution of students across three main fields (medical and health sciences, technical/science, and humanities/social sciences). However, this sampling approach may limit the representativeness of the sample for the overall population of Polish university students, potentially constraining the generalizability of the findings. Due to the cross-sectional nature of our study, it is not possible to establish the direction of the relationship between the variables studied. Furthermore, it is important to note that the study relied on self-reported data and did not involve clinical confirmation of either depression or internet addiction. This study employed the BDI-I questionnaire because the Polish version of the BDI-II was not available at the time of data collection. Compared with the BDI-II, the BDI-I may be less sensitive to somatic symptoms of depression, and in this respect depressive symptoms in our study may be underestimated. Although the BDI-I remains a widely used and validated instrument, this limitation should be taken into account when interpreting the results. The Health Behaviour Inventory, which is commonly used in Poland, was selected to measure health behaviour. It has the advantage of providing a comprehensive evaluation of health behaviours and using sten norms for the Polish population. However, it is important to note that its subcategories have relatively low reliability (ranging from 0.60 to 0.65). Furthermore, the questionnaire does not include a direct question regarding the amount of time spent on physical activity. Such a question should be considered in the future to better compare the results obtained with those of other authors. The study addressed the potential for multicollinearity among explanatory variables, which was automatically verified by the regression model. Only the specific IZZ subfactors were strongly correlated with each other and collinearly related to the overall score (consistent with the theoretical assumption, the factors measured specific manifestations of general health-related behaviors). Therefore, the model used only factor scores that, although correlated, were not identical. Among the remaining variables, loneliness correlated with depressive symptoms at a moderately weak level, while the overall IZZ score was not significantly associated with either loneliness or depressive symptoms (the strength of the correlation is presented in Fig. 1).

## Conclusions

Our results show that there is a correlation between PIU and negative outcomes, such as loneliness, depressive symptoms, and a decrease in positive health behaviours (overall and in the categories of eating habits, preventive behaviour, positive mental attitude, and health practices). Loneliness was identified as a positive predictor of PIU, while depression symptoms and a positive mental attitude were found to be negative predictors of PIU. Other health behaviors were not associated with PIU.

These results suggest an association between positive mental attitude, loneliness, depressive symptoms, and PIU. Further research is needed to establish the direction of this relationship. Prospective studies may confirm that attention to mental wellbeing and mental health promotion can provide prevention of PIU, in addition to the benefits known to date.

## Data Availability

The data presented in this study is available on request from the corresponding author.

## Abbreviation

PIU: Problematic Internet Use Test
DJGLS: De Jong Gierveld Loneliness Scale
BDI I: Beck Depression Inventory I
HBI: Health-Related Behaviour Inventory
